# Rewiring Tumor Lifelines: Translating Hypoxia- and Pseudohypoxia-Driven Angiogenesis into Therapeutic Breakthroughs

**DOI:** 10.3390/cells15141295

**Published:** 2026-07-20

**Authors:** Michael Boulis, Fady Tawfik, Anitha Kota Shenoy

**Affiliations:** 1 Naresh K. Vashisht College of Medicine, Texas A&M University, Bryan, TX 77807, USA; mboulis@tamu.edu; 2 College of Medicine, Howard University, Washington, DC 20059, USA; fady.tawfik@bison.howard.edu; 3 Department of Medical Education, Naresh K. Vashisht College of Medicine, Texas A&M University, Bryan, TX 77807, USA

**Keywords:** hypoxia, pseudohypoxia, tumor angiogenesis, hypoxia-inducible factors, VHL mutations, SDH mutations, VEGF, anti-angiogenic therapy, HIF inhibitors, clinical trials

## Abstract

**Highlights:**

**What are the main findings?**

- Hypoxia and pseudohypoxia, the latter driven by *VHL*, *SDH*, or *FH* mutations, converge on HIF-α stabilization to promote tumor angiogenesis, metabolic rewiring, and immune evasion.
- Tumors access a blood supply through multiple, often anti-angiogenic-resistant routes, including sprouting angiogenesis, vessel co-option, and vascular mimicry.

**What are the implications of the main findings?**

- Targeting the HIF-α hub and its resistance pathways—HIF-2α inhibitors, hypoxia-activated prodrugs, metabolic modulation, and anti-angiogenic/immunotherapy combinations—offers strategies to overcome anti-angiogenic resistance.
- Biomarker-guided selection (*VHL*/*SDH* mutational status, CAIX expression, and hypoxia PET) can match patients to hypoxia-directed therapies.

**Abstract:**

Hypoxia and the evolving concept of pseudohypoxia are critical in driving tumor angiogenesis, contributing to malignancy progression and therapeutic resistance. Angiogenesis, a common feature of many solid tumors, is promoted by hypoxia-induced overexpression of pro-angiogenic factors (e.g., VEGF, FGF) and genetic mutations (e.g., *VHL*, *SDH*) that stabilize hypoxia-inducible factors (HIF) even in normal oxygen conditions, a phenomenon known as pseudohypoxia. Recent experimental studies challenge the view that hypoxia universally enhances vessel growth. In certain models, severe oxygen deprivation impairs angiogenesis. Furthermore, tumor-mediated metabolic reprogramming can drive immune evasion via HIF stabilization in immune cells. These paradoxes, together with persistent therapy resistance and the limited effectiveness of current anti-angiogenic treatments, reveal critical gaps in our understanding of how hypoxic signaling modulates vascular and immune dynamics within the tumor microenvironment. These complexities demand more detailed exploration of underlying processes and the development of innovative therapeutic strategies. Here, we review recent mechanistic studies on tumor angiogenesis, summarizing therapeutic and diagnostic advances from both preclinical and clinical studies. We further discuss strategies to exploit hypoxic vulnerabilities, including HIF inhibitors, hypoxia-activated prodrugs, vascular normalization, combination regimens to restore immunity, biomarker-guided patient selection, and advanced hypoxia-targeted imaging to improve outcomes in angiogenesis-driven cancers.

## 1. Introduction

Angiogenesis, the formation of new blood vessels from pre-existing vessels, and vasculogenesis, the de novo generation of vessels from endothelial progenitors, are fundamental processes in both physiology and disease [1]. They are critical during embryonic development [2] and tissue repair [3]. They also contribute to pathological conditions such as ischemic heart disease [4], diabetic retinopathy [5], rheumatoid arthritis [6], and chronic inflammation [7]. Among these, tumor angiogenesis, the formation of new blood vessels from existing vasculature, is one mechanism within the broader hallmark of inducing or accessing vasculature [8], enabling tumor growth, metastasis, and resistance to therapy. A major driver of this process is the hypoxic tumor microenvironment, which arises as proliferating cancer cells outgrow the local oxygen supply [9,10]. In conditions of low oxygen availability, stabilization of the transcription factor hypoxia-inducible factor–1α (HIF-1α) activates the transcription of hundreds of genes involved in angiogenesis, including vascular endothelial growth factor (*VEGF*), thereby promoting cellular adaptation to stress [9,11]. Apart from angiogenesis, hypoxic signaling mediated by HIF transcription factors promotes tumor cell survival, drives metabolic reprogramming and an immunosuppressive tumor microenvironment [10,12], contributing to aggressive tumor phenotypes. In fact, roughly half of all solid tumors are hypoxic at any given time [9]. Notably, however, hypoxia is patchy and variable. Many tumors or early-stage lesions are not overtly oxygen-deprived. In these cases, a “pseudohypoxic” phenotype can emerge, in which hypoxia-responsive pathways are activated despite adequate oxygen tension [13,14]. Such pseudohypoxia arises from genetic and metabolic alterations, including loss of von Hippel–Lindau (*VHL*) gene or mutations in metabolic enzymes like succinate dehydrogenase (SDH) and fumarate hydratase (FH) enzymes that inhibit HIF-1α degradation, thereby mimicking a true hypoxic signaling [15,16,17]. Here, we review recent advances in understanding how hypoxia and pseudohypoxia converge on angiogenic programs in cancer, shape tumor vascular dynamics and metabolism, and highlight clinical trials of targetable pathways with potential to improve treatment efficacy and overcome resistance.

## 2. Tumor Angiogenesis

Early pioneers recognized that rapidly growing cancers are highly vascular while dormant lesions are not [18]. In fact, Judah Folkman’s seminal work established that solid tumors beyond 1–2 mm^3^ become hypoxic and must induce angiogenesis to progress, thus establishing the foundation for the concept of angiogenesis as a therapeutic target in cancer [10,18,19]. This classical model, though foundational, has since been refined: updated hallmark frameworks recognize that not all tumors depend on angiogenesis, as some progress by co-opting existing vasculature [8].

Within growing tumors, regions of low oxygen (often defined as <10 mmHg pO2) develop when cell proliferation exhausts the supply of existing vasculature. This hypoxia stabilizes HIF-1α subunits (which are otherwise rapidly degraded in normoxia) and triggers a concerted pro-angiogenic transcriptional program [9,11]. This marks the angiogenic switch, the point at which pro-angiogenic signals overwhelm endogenous inhibitors such as thrombospondin-1 and angiostatin, allowing previously quiescent vessels to begin sprouting [20]. The HIF heterodimer binds hypoxia response elements (HREs) in target genes, upregulating VEGF-A and other soluble factors that drive endothelial cell proliferation [9,11]. VEGF/VEGFR-2 signaling is central to angiogenesis, stimulating endothelial cell proliferation, migration, and survival through downstream pathways such as PI3K/AKT/mTOR and Raf/MEK/ERK [21,22]. This pathway was the first to be therapeutically targeted in angiogenesis, paving the way for the exploration of several additional targets [23]. Similarly, FGF/FGFR signaling contributes by inducing matrix metalloproteinase (MMP) secretion, extracellular matrix remodeling, and endothelial cell differentiation via multiple downstream effectors [24,25]. In lung cancers that harbor activating *EGFR* mutations, EGFR signaling has been shown to upregulate VEGF expression and promote endothelial cell proliferation, thereby enhancing angiogenic responses [26]. Aberrant activation of the FGF/FGFR and EGFR/ERK pathways are shown as key drivers of angiogenesis in specific tumors, including pleuropulmonary blastoma and salivary gland cancers [27,28,29]. PDGF signaling also plays a crucial role by supporting vessel maturation and facilitates recruitment of pericytes, essential for vessel stability [30].

Unlike the tightly coordinated angiogenesis seen during embryogenesis or wound healing, tumor-derived vessels are disorganized, leaky and poorly supported by pericytes, contributing to therapy evasion [31]. These structural defects elevate interstitial pressure, impede drug and immune-cell delivery, and aid metastasis [31]. Expanding upon the structural abnormality of the tumor blood vessels, several single-cell transcriptomic studies have revealed the heterogeneity of endothelial cell populations within the tumor in both patient-derived samples and mouse models [32]. A pan-cancer endothelial cell atlas constructed by Li et al. demonstrated that specific endothelial subpopulations, such as CXCR4+ tip cells, are associated with invasive vessel sprouting and are negatively correlated with responsiveness to anti-angiogenic therapies [33]. Conversely, the presence of E-selectin expressing (SELE+) venous endothelial cells has been linked with improved leukocyte adhesion and T-cell infiltration, which correlated with better response to immunotherapy [34].

Like the heterogeneity observed in endothelial populations during angiogenesis, the process of new blood vessel formation is highly variable and arises through several distinct mechanisms [35]. These include classical sprouting angiogenesis (endothelial tip cells forming new capillary sprouts) [36], intussusceptive angiogenesis (a vessel splitting in two) [37], ansiform angiogenesis (loop formation) [37], vasculogenesis (de novo vessel formation from endothelial progenitor cells during development) [38], recruitment of circulating endothelial progenitor cells, vascular mimicry (cancer cells forming matrix-lined channel networks) [39], and even transdifferentiation of cancer stem cells into endothelial-like cells, itself a form of vascular mimicry, [40]. Tumors can also use pre-existing vessels (called vessel co-option), bypassing new vessel growth entirely, demonstrating that angiogenesis is not obligatory for tumor progression [41]. These alternative vascularization strategies are well illustrated in glioblastoma, where malignant stem cells express endothelial markers (e.g., CD31, CD34, vWF) and form tube-like networks in vitro [42]. This vascular plasticity, the capacity to switch between sprouting angiogenesis, vascular mimicry, and vessel co-option, means that anti-angiogenic drugs often fail despite dozens of FDA-approved VEGF-pathway inhibitors, and tumors rapidly evolve resistance by switching to alternate vascularization modes or co-opting existing vasculature [23].

Overall, the mechanisms underlying angiogenesis, angiogenic processes, as well as the composition of endothelial cell populations are highly variable and context-dependent within tumors. This cellular and molecular heterogeneity not only complicates effective therapeutic targeting but also promotes immune evasion, metabolic reprogramming and metastasis, which further contribute to treatment resistance. Thus, it is essential to revisit and refine our understanding of angiogenesis considering both past and emerging advances, with particular attention to unique microenvironmental factors, especially hypoxia and pseudohypoxia that function as key regulators of angiogenic, metabolic and immune processes.

## 3. Tumor Angiogenesis Driven by Hypoxia and Pseudohypoxia

Oxygen deprivation is a primary trigger of angiogenesis. Under hypoxia, cells upregulate glucose transporters (GLUT) and glycolytic enzymes to compensate, producing lactate and acidifying the microenvironment [9]. This acidification is not merely a byproduct but actively contributes to tumor progression. It promotes angiogenesis by stimulating pro-angiogenic factors such as VEGF and facilitates local invasion by remodeling the extracellular matrix [43,44,45]. Immunohistochemical studies have demonstrated that invasive tumor edges express high levels of GLUT1 and acid-extruding transporters, correlating increased invasion with regions of low pH [46].

When tumors outgrow their blood supply, HIF transcription factors stabilize and drive forward a pro-angiogenic cascade [18]. Upon stabilization, it transactivates VEGF-A, FGF2, PDGF, and matrix-modifying enzymes. Mutations in oxygen-sensing genes mimic this state. Loss of VHL protein, as seen in clear cell renal cell carcinoma (ccRCC) and certain paragangliomas, prevents HIF-1α degradation even in normoxia, leading to pseudohypoxic transcriptional programs [14,18]. Likewise, loss of TCA-cycle enzymes (SDH, fumarase) causes accumulation of succinate/fumarate that block prolyl-hydroxylases, again stabilizing HIFs [14]. These genetic alterations thus bypass normal oxygen dependence, ensuring persistent VEGF secretion and angiogenesis [14,18].

### 3.1. Hypoxia

Under true hypoxia, HIF-1α (and to a lesser extent HIF-2α) mediates angiogenic signaling [18]. HIF-1α, when activated under low oxygen conditions, is crucial for regulating the downstream gene VEGF. HIF-1α stabilization increases VEGF and FGF transcription by greater than 2-fold in many tumors [18]. Seminal experiments by Forsythe et al. elucidated the molecular mechanism underlying this process by cloning the human VEGF promoter and identifying a hypoxia-response element (HRE) that binds HIF-1 under low oxygen conditions [47]. This binding activates VEGF transcription, establishing the canonical HIF-VEGF axis that drives vascular proliferation within tumors. Beyond this canonical axis, the transcription factor WT1 (Wilms tumor 1) provides a further layer of angiogenic control. Acting downstream of and together with HIF-1, WT1 directly activates VEGF transcription and, through the splicing factors SRPK1 and SRSF1, shifts the balance of VEGF isoforms toward pro-angiogenic forms [48,49,50]. WT1 also coordinates tumor vascularization with immune modulation and metastasis, contributing to an immunosuppressive, myeloid- and MDSC-rich pro-angiogenic microenvironment [49]. Moreover, WT1 is a leading tumor immunotherapy target: multivalent WT1 peptide vaccines such as galinpepimut-S have shown immunogenicity and clinical activity in completed and ongoing trials, including in combination with immune-checkpoint blockade [51]. The critical importance of VEGF dosage for angiogenesis was confirmed through genetic loss-of-function studies, which demonstrated that VEGF haploinsufficiency results in embryonic lethality due to defective vasculature, highlighting the central role of VEGF in both normal development and tumor angiogenesis [52,53]. Ever since these foundational discoveries, we have come a long way in understanding the breadth and complexity of hypoxia-driven tumor biology [54]. We now recognize that HIF-1α stabilization occurs through multiple oxygen-independent mechanisms that include kinase-mediated phosphorylation [55] and metabolic rewiring [56].

Beyond transcriptional activation, hypoxia influences tumor metabolism by reprogramming cells toward glycolysis and glutamine metabolism, thus, accommodating energy production in low-O_2_ [9]. The shift towards glycolysis is known as the Warburg effect [57,58]. Under hypoxia, HIF-1α upregulates a broad array of glycolytic genes, including *SLC2* for GLUTs, hexokinase 1/2 (*HK1/HK2*), phosphofructokinase 1 (*PFK1*), phosphoglyceride kinase-1 (*PGK1*), 6-phosphofructo-2-kinase/fructose-2,6-bisphosphatase (*PFKFB3*) and *LDHA*, thereby driving glycolysis and reducing mitochondrial oxidative metabolism [59]. In line with this, pharmacologic or molecular inhibition of endothelial PFKFB3 has been shown to normalize tumor vasculature, suppress pathologic angiogenesis and metastasis, and enhance antitumor efficacy of chemotherapy by disrupting the glycolytic support for vessel sprouting and tumor progression [60,61,62]. Together, these individual findings indicate that hypoxia-induced metabolic reprogramming is a potential target to counter tumor growth and angiogenesis to improve therapeutic response.

Hypoxia also profoundly modulates microRNA (miRNA) expression [63]. For example, miR-210 is induced by hypoxia and regulates endothelial cell function and angiogenic gene expression, fine-tuning the vascular response and tumor-immune interactions [64]. In this context of tumor cell–immune cell communication, exosomes play a key role by transferring bioactive cargos such as mRNAs, including VEGFA mRNA, as well as miR-210 and proteins like HIF-1α [65]. Thus, hypoxia enhances microRNA (miR-210) and exosome release, modulating angiogenic signaling and intercellular communication within the tumor microenvironment [66]. Hypoxia further remodels the immune microenvironment by recruiting immunosuppressive cells such as tumor-associated macrophages (TAMs) and myeloid-derived suppressor cells (MDSCs) [67]. These cells secrete pro-angiogenic factors including VEGF and matrix metalloproteinases (MMPs), which promote extracellular matrix remodeling and tumor vascularization, collectively supporting tumor progression [68].

A number of hypoxia-induced cellular events, including the above-mentioned mechanisms, increase tumor resilience and therapeutic resistance [66]. Hypoxia activates stress-adaptation pathways such as the unfolded protein response (UPR) and mTOR signaling, which support hypoxia-driven angiogenesis by promoting vascular remodeling [12]. HIFs influence epigenetic regulation within tumors [69], where they affect chromatin remodeling, DNA methylation, and histone modifications, regulating gene expression programs critical for angiogenesis, tumor progression, and therapy resistance [70]. For instance, hypoxic conditions alter the activity of demethylases and histone-modifying enzymes affecting genes like *VEGF* and tumor suppressors such as *BRCA1* [71]. These hypoxia-induced genetic and epigenetic reprogramming further increase tumor cell plasticity, enabling adaptation to fluctuating oxygen levels and resistance to therapy [72,73]. Together, these processes create a feedback loop: angiogenesis is stimulated to relieve hypoxia, but disorganized vasculature sustains low-oxygen regions and immunosuppression [57,58,74].

### 3.2. Pseudohypoxia

In contrast, pseudohypoxia refers to HIF stabilization without a true oxygen deficit. Mechanistically, pseudohypoxia arises from genetic or metabolic disruptions that stabilize HIF-α subunits independently of oxygen tension. A classic example is loss-of-function mutations in the *VHL* gene, as seen in VHL syndrome, preventing the ubiquitin-mediated degradation of HIF-α and resulting in constitutive HIF activation [14,75,76,77]. In VHL-deficient cells, HIF-α accumulates and constitutively activates the hypoxia program even in the presence of normal oxygen levels. This mechanism has been verified through protein interaction studies and functional rescue experiments [13].

Central to this process is the requirement of prolyl-hydroxylation (PHD) of HIF-α, which serves as the molecular gate for VHL-mediated proteasomal degradation [78,79,80]. Ivan et al. demonstrated that oxygen and iron are necessary for this modification [81]. When PHD activity is lost or inhibited due to hypoxia or iron deficiency, HIF-α is no longer hydroxylated. This disrupts its turnover, leading to accumulation and stabilization of HIF-α. This stabilization effectively mimics hypoxia and subsequently activates proangiogenic genes [81].

Metabolic defects in the tricarboxylic acid (TCA) cycle also contribute to pseudohypoxia. Similar to how mutations in *VHL* and *PHD* disrupt HIF regulation, mutations in TCA cycle enzymes genes such as *SDH* and *FH*, lead to accumulation of oncometabolites like succinate or fumarate, which inhibit PHDs and stabilize HIF-α, further promoting a pseudohypoxic state [13,14,75,77,82].

In these contexts, HIF-2α often predominates, particularly in *VHL*- and *SDH*-mutant tumors [75,77]. Similar to true hypoxia, pseudohypoxia also induces a metabolic shift toward aerobic glycolysis (the Warburg effect), with increased expression of glycolytic enzymes and pyruvate dehydrogenase kinase (PDK) [13,82,83].

The Warburg effect increases resistance to apoptosis in tumor cells by reducing mitochondrial activity, which leads to decreased cytochrome c release, a critical trigger for the intrinsic apoptotic pathway, and by altering the expression of apoptotic regulators, including upregulation of anti-apoptotic Bcl-2 family proteins and downregulation of pro-apoptotic factors. This metabolic reprogramming, characterized by a shift from oxidative phosphorylation to aerobic glycolysis, limits mitochondrial outer membrane permeabilization and cytochrome c efflux, thereby suppressing caspase activation and apoptosis initiation [84,85,86,87,88,89].

Additionally, the glycolytic phenotype is associated with increased intracellular pH and reduced reactive oxygen species (ROS) production, both of which further inhibit apoptosis [84,88,89]. The upregulation of glycolytic enzymes and PDK by HIFs and oncogenic signaling inhibits pyruvate entry into mitochondria, reinforcing mitochondrial quiescence and resistance to apoptotic stimuli [86,89]. These metabolic adaptations also promote the expression of anti-apoptotic proteins such as Bcl-2 and Bcl-xL, while suppressing pro-apoptotic proteins like Bax and Bak, further establishing apoptosis resistance [84,88,89]. This metabolic rewiring parallels hypoxic effects. Therapeutically, targeting metabolic enzymes, restoring normal mitochondrial function, or modulating PHD activity are promising strategies in cancers driven by pseudohypoxia.

#### Genetic Triggers and Their Associated Tumor Spectra

Two genetic lesions account for most pseudohypoxic tumors, and each carries a characteristic tumor spectrum.

**VHL mutations.** Building on the VHL and PHD mechanisms described above, loss-of-function VHL mutations abolish oxygen-dependent HIF-α degradation. HIF-α accumulates constitutively, driving transcription of *VEGF*, *EPO*, *PDGF*, and *cyclin D1* even under normoxic conditions [90,91]. *VHL* inactivation occurs in up to 90% of clear cell renal cell carcinomas (ccRCC), producing their characteristic hypervascular phenotype [92]. Germline *VHL* mutations cause VHL syndrome, which predisposes to CNS and retinal hemangioblastomas, pancreatic neuroendocrine tumors, pheochromocytomas, paragangliomas, endolymphatic sac tumors, and epididymal cystadenomas [93,94].

Loss-of-function in any SDHx subunit (SDHA–D) impairs conversion of succinate to fumarate; the resulting succinate accumulation competitively inhibits PHD enzymes and stabilizes HIF-α [95,96]. SDH-deficient tumors overexpress HIF-1α, activate VEGF, and display high microvessel density [49]. *SDHx* mutations account for approximately 30–40% of hereditary pheochromocytoma and paraganglioma cases, with *SDHB* mutations carrying the highest metastatic risk [97,98]; the spectrum also includes SDH-deficient (wild-type) gastrointestinal stromal tumors, renal cell carcinoma, and pituitary adenomas [97,99].

Although, *VHL*- and *SDH*-mutations arise through distinct upstream mechanisms, they converge on a shared pseudohypoxic transcriptional program. Both promote hypoxia signaling, angiogenesis, and oxidoreductase imbalance. However, *VHL*-mutant tumors tend to show preferential HIF-2α activation whereas *SDH*-mutant tumors exhibit relatively greater HIF-1α expression [100,101].

Representative studies of pseudohypoxia-driven angiogenesis are summarized in Table 1.

### 3.3. Overlapping Pathways

Regardless of origin, hypoxia and pseudohypoxia converge on core pathways. Central is HIF-α stabilization, which broadly orchestrates angiogenesis, metabolism and immune modulation [14,18,73,102,103,104]. Importantly, HIF stabilization is not restricted to angiogenic tumors; it is also detected in non-angiogenic, vessel co-opting tumors [92]. Under both conditions, tumors adopt aerobic glycolysis. HIF upregulates GLUT1 and HK to boost glucose uptake and increases glutaminase expression for glutamine use in the TCA cycle [105]. Simultaneously, HIFs promote extracellular matrix remodeling through MMP upregulation and enhancing cell motility, enabling invasion into adjacent vasculature. On the immune front, stabilized HIF-1α induces VEGF and PD-L1, limiting T-cell infiltration and recruiting regulatory immune cells [12]. In effect, hypoxic signaling and immune checkpoint pathways reinforce each other. HIF-driven angiogenesis creates physical and chemical barriers, while immune suppression prevents vessel pruning by the immune system.

**Figure 1:**
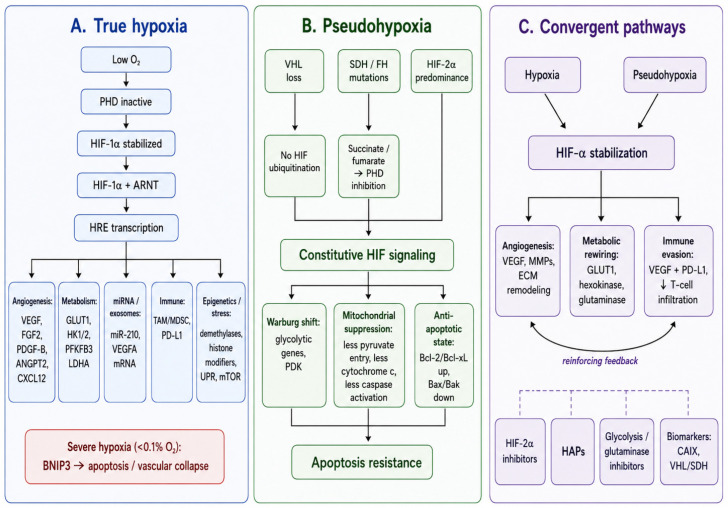
**Mechanisms of hypoxia- and pseudohypoxia-driven tumor signaling.** (**A**) Under true hypoxia, reduced oxygen availability inhibits prolyl hydroxylase domain enzymes, stabilizing HIF-1α and enabling HIF-1α/ARNT-mediated transcription of angiogenic, metabolic, immune, microRNA/exosomal, and epigenetic programs; severe hypoxia may instead promote BNIP3-mediated apoptosis and vascular collapse. (**B**) Pseudohypoxia results from oxygen-independent HIF stabilization through VHL loss, SDH/FH mutations with succinate or fumarate accumulation, and HIF-2α predominance, promoting glycolytic reprogramming and resistance to apoptosis. (**C**) Both pathways converge on HIF-α stabilization, driving angiogenesis, metabolic rewiring, and immune evasion while creating therapeutic vulnerabilities to HIF-2α inhibitors, hypoxia-activated prodrugs, metabolic inhibitors, and biomarker-guided treatment. Abbreviations: ARNT, aryl hydrocarbon receptor nuclear translocator; CAIX, carbonic anhydrase IX; ECM, extracellular matrix; FH, fumarate hydratase; HAP, hypoxia-activated prodrug; HIF, hypoxia-inducible factor; HRE, hypoxia-response element; MDSC, myeloid-derived suppressor cell; MMP, matrix metalloproteinase; PHD, prolyl hydroxylase domain enzyme; SDH, succinate dehydrogenase; TAM, tumor-associated macrophage; UPR, unfolded protein response; VHL, von Hippel–Lindau. Created in BioRender. https://BioRender.com/7crp81z.

This intimate crosstalk supports combined targeting strategies. For instance, dual blockade of VEGF and HIF-α signaling may prevent compensatory upregulation of alternate angiogenic factors. Likewise, pairing metabolic inhibitors (e.g., glutaminase or glycolysis inhibitors) with anti-angiogenics could starve tumors of hypoxic adaptation routes. Biomarkers of hypoxia pathways, including CAIX expression and *VHL/SDH* mutational status, can help identify patients most likely to benefit from HIF2α inhibitors or hypoxia-activated prodrugs. The convergence of these pathways and their corresponding therapeutic vulnerabilities are summarized in Figure 1.

**Table 1:** Representative studies of pseudohypoxia-driven angiogenesis. Studies are organized by tumor type or experimental model and the underlying pseudohypoxic mechanism (VHL loss, impaired PHD activity, SDH or FH mutations, and mitochondrial oxygen-sensing dysfunction), with key angiogenic findings and clinical or translational relevance. Abbreviations: ccRCC, clear cell renal cell carcinoma; CNS, central nervous system; FH, fumarate hydratase; HIF, hypoxia-inducible factor; PDK, pyruvate dehydrogenase kinase; PHD, prolyl hydroxylase domain protein; pNET, pancreatic neuroendocrine tumor; SDH, succinate dehydrogenase; TCA, tricarboxylic acid; TKI, tyrosine kinase inhibitor; VEGF (R), vascular endothelial growth factor (receptor); VHL, von Hippel–Lindau.

Study	Tumor Type or Model	Pseudohypoxic Mechanism	Key Angiogenic Findings	Clinical or Translational Relevance	Ref.
Ivan et al. (2001)	In vitro models of HIF-α regulation	Loss of prolyl hydroxylase–dependent HIF-α hydroxylation and VHL recognition	Oxygen- and iron-dependent prolyl hydroxylation is required for VHL-mediated HIF-α ubiquitination and degradation; impaired hydroxylation permits constitutive HIF stabilization and activation of proangiogenic genes, including VEGF.	Established the molecular basis of oxygen sensing and pseudohypoxic HIF activation caused by impaired PHD or VHL function.	[81]
Bratslavsky et al. (2007)	Clear cell renal cell carcinoma	VHL loss of function	VHL inactivation prevented HIF-α degradation under normoxia, resulting in persistent HIF signaling, VEGF production, and angiogenic pathway activation.	Provided a mechanistic rationale for targeting HIF-α and VEGF-dependent signaling in VHL-deficient ccRCC.	[15]
Hayashi et al. (2019)	Multiple cancer types; review	VHL loss, PHD inhibition, and metabolic-enzyme alterations	Characterized oxygen-independent HIF-1α activation; VHL-deficient cells constitutively accumulate HIF-α and activate angiogenic transcriptional programs.	Provided a framework for distinguishing true hypoxia from pseudohypoxia and for selecting mechanism-specific therapies.	[13]
Kluckova and Tennant (2018)	Pheochromocytoma and paraganglioma	SDH or FH mutation with succinate/fumarate accumulation and PHD inhibition	Oncometabolite accumulation inhibited PHD activity, stabilized HIF-α, and sustained VEGF-mediated angiogenic signaling; tumors may develop dense but functionally inefficient vascular networks.	Highlighted metabolic vulnerabilities and supported targeting oncometabolite production, mitochondrial metabolism, or HIF regulation.	[14]
Favier and Gimenez-Roqueplo (2010)	Pheochromocytoma and paraganglioma	SDH-associated pseudohypoxia	SDH-mutant tumors showed constitutive HIF activation and increased angiogenic mediators, producing highly vascular but inefficiently perfused networks.	Supported pseudohypoxia as a unifying mechanism in hereditary pheochromocytoma and paraganglioma.	[77]
Celada et al. (2023)	Paraganglioma and pheochromocytoma	SDH mutation–associated pseudohypoxic phenotype	Pseudohypoxic tumors exhibited an immunosuppressive phenotype in which HIF-regulated angiogenic and immune signaling promoted aberrant vascularization and impaired antitumor immunity.	Linked pseudohypoxic angiogenesis with immune evasion and supported combined angiogenesis- and immune-directed therapies.	[106]
Wu et al. (2021)	Pulmonary hypertension and cancer models	Mitochondrial dysfunction and altered oxygen-sensing/redox signaling	Mitochondrial abnormalities altered reactive oxygen species and oxygen-sensing pathways, producing oxygen-independent HIF stabilization and angiogenic gene expression.	Expanded the pseudohypoxia concept beyond VHL, SDH, and FH mutations to include mitochondrial oxygen-sensing dysfunction.	[82]
Mohlin et al. (2017)	Multiple tumor types; review	VHL, SDH, and FH alterations	Pseudohypoxia produced a persistent HIF-activated state under normoxia, sustaining glycolysis, angiogenic signaling, and impaired differentiation; HIF-2α may predominate in selected *VHL*- and *SDH*-mutant tumors.	Highlighted pseudohypoxia in maintaining undifferentiated, proangiogenic tumor phenotypes.	[75]
Paredes et al. (2021)	Multiple cancer types	TCA-cycle dysfunction and metabolic adaptation	Pseudohypoxic reprogramming promoted aerobic glycolysis, PDK expression, reduced mitochondrial oxidative metabolism, and persistent HIF-dependent survival and angiogenic signaling.	Supported strategies to restore mitochondrial function or target pseudohypoxic metabolic dependencies.	[76]
Jonasch et al. (2021), MK-6482-004	VHL-associated ccRCC, pNET, and CNS hemangioblastomas	VHL loss with constitutive HIF-2α activation	The HIF-2α inhibitor belzutifan produced substantial responses across VHL-associated hypervascular tumors by suppressing HIF-2α and downstream VEGF signaling.	Clinical validation that direct HIF-2α inhibition can produce durable regression; supported approval of belzutifan.	[93]
Choueiri et al. (2025), LITESPARK-003	First-line metastatic ccRCC	VHL loss with constitutive HIF-2α activity	Belzutifan plus the VEGFR TKI cabozantinib achieved a 70% objective response rate (8% complete responses) via simultaneous HIF-2α and VEGF blockade.	Demonstrated benefit of combining pseudohypoxia inhibition with antiangiogenic therapy in VHL-driven tumors.	[107]

## 4. Hypoxia and Pseudohypoxia in Tumor Vascular Dynamics

Hypoxia, an inadequate oxygen supply within tumor regions, and pseudohypoxia, a pathological HIF activation in normoxia due to genetic aberrations such as *VHL* loss and *SDH* mutation or metabolic disruptions, profoundly influence tumor vascular dynamics. In oxygen-starved zones, HIF-α stabilization upregulates pro-angiogenic mediators (notably VEGF), driving exuberant but disorganized neovascularization [12]. Pseudohypoxic tumors similarly trigger HIF-driven angiogenic programs in the absence of true hypoxia, effectively “rewiring” vascular growth signals even in well-oxygenated tissue [106]. Both conditions converge to induce aberrant vessel remodeling and metabolic adaptation. The resulting nascent vessels are tortuous, hyperpermeable, and poorly perfused, failing to effectively oxygenate the tumor bed [12]. Ironically, extreme or prolonged oxygen deprivation can destabilize angiogenesis. Severe hypoxia impairs endothelial cell function and vessel integrity in certain models, attenuating rather than enhancing vascular expansion [108]. Furthermore, hypoxic and pseudohypoxic signaling drives a shift toward glycolytic metabolism and activates survival pathways, changes that promote resistance to therapy-induced stress. Hypoxic tumor cells often enter a quiescent, therapy-tolerant state, undermining the efficacy of chemotherapy and radiotherapy [109,110]. These microenvironmental conditions also foster immune escape: HIF-mediated factors (e.g., VEGF, adenosine, PD-L1) recruit immunosuppressive myeloid cells and impede cytotoxic lymphocytes, creating a permissive niche that reinforces pathological angiogenesis [12,106]. In aggregate, hypoxia and pseudohypoxia orchestrate vascular remodeling that can both promote new vessel growth and diminish vessel function, highlighting the two-faced nature of hypoxic signaling in tumors.

### 4.1. Dual Roles of Hypoxic Signaling

In moderate hypoxia, HIF-1α accumulation launches a potent transcriptional burst that accelerates endothelial recruitment and vessel sprouting. VEGF-A induction is the most visible outcome of this shift, but genomic profiling shows that hypoxic regions simultaneously elevate FGF2, PDGF-B, angiopoietin 2 (ANGPT2), CXCL12, and a broader set of stromal-modulating mediators [111,112]. Quantitative analyses of patient tumors reinforce the strength of this program: HIF-1α expression correlates with VEGF levels (r ≈ 0.59) and with microvessel density (r ≈ 0.53, *p* < 0.005), confirming that hypoxic signal intensity tracks closely with angiogenic output [18].

Yet the same molecular machinery becomes destabilizing as oxygen levels fall further. When O_2_ tension drops below roughly 0.1%, a threshold recorded in deeply hypoxic tumor cores, endothelial cells enter a stress state characterized by mitochondrial dysfunction, heightened ROS exposure, and rising susceptibility to programmed cell death [103,113]. Under these conditions, HIF-1α transitions into a pro-death regulator, upregulating genes such as BNIP3 that trigger apoptosis in both endothelial and tumor compartments [103,114]. Instead of generating functional vasculature, the hypoxic microenvironment collapses into necrotic zones, leaving only fragmented, non-perfused vessels at the perimeter. In murine breast tumors, microvessels bordering anoxic tissue show an abrupt loss of perfusion and structural integrity, and vessel density in these peri-necrotic rims correlates strongly with the extent of regional necrosis (Spearman ρ ≈ 0.83), indicating that extreme hypoxia produces excessive, dysmorphic angiogenesis that fails to restore circulation [113].

This shift from constructive to destructive signaling is sharpened by the divergent roles of HIF-1α and HIF-2α. HIF-1α dominates acute hypoxia, activating glycolytic adaptation and rapidly mobilizing sprouting responses, whereas HIF-2α is expressed in a more restricted, tissue-specific set of cell types (including vascular endothelium and renal and hepatic cells) and is associated with responses to more prolonged hypoxia, such as erythropoietin induction and vascular remodeling [103]. Their transcriptional targets are distinct but partially overlapping, contributing to a division of labor: HIF-1α initiates angiogenesis; HIF-2α, when present in appropriate physiological gradients, can refine it. However, when this choreography is disrupted, either by severe hypoxia that sustains HIF-1α activity beyond its adaptive window or by pseudohypoxic mutations that elevate HIF-2α in the absence of spatial oxygen cues, vascular development becomes uncoordinated.

Pseudohypoxia intensifies this disruption by uncoupling hypoxic signaling from oxygen tension entirely. VHL-deficient and *SDH/FH*-mutant tumors stabilize HIF complexes constitutively, producing angiogenic landscapes that resemble profoundly hypoxic tissue despite adequate perfusion [23,77]. In ccRCC, constitutive HIF-2α due to VHL inactivation increases expression of pro-angiogenic genes such as *VEGF*, resulting in a hypervascular phenotype and increased microvessel density. However, HIF-2α–driven VEGF and ANGPT2 promote sprawling but fragile networks marked by poor pericyte coverage and chaotic branching [115,116]. Succinate-accumulating paragangliomas exhibit a similar dichotomy: dense capillary networks that appear exaggerated yet deliver surprisingly inefficient perfusion [77]. These observations highlight that hypoxic signaling, when deployed without spatial control, drives angiogenesis quantitatively while degrading it qualitatively.

The most clinically consequential aspect of this duality emerges when hypoxia interacts with therapy. Anti-VEGF agents heighten intratumoral hypoxia by pruning vessels, and the resulting intensification of HIF signaling frequently triggers compensatory surges of FGF2, PDGF-BB, IL-17 and PlGF, enabling residual tumor cells to rebuild vasculature through alternative routes [117,118,119,120]. Angiogenesis is thus not simply restored; it becomes more heterogeneous, more reliant on non-VEGF pathways, and more resistant to inhibition. In this sense, hypoxia serves as both the initiating force behind tumor vascularization and the engine of resistance that erodes the efficacy of anti-angiogenic therapy.

### 4.2. Immune Evasion and Metabolic Rewiring

The hypoxic tumor microenvironment further amplifies angiogenic complexity through the regulatory effect of immune cells. Both hypoxia and pseudohypoxia modulate the immune microenvironment by recruiting immunosuppressive cells such as TAMs and MDSCs [121]. Immune interactions influence angiogenesis, while abnormal tumor vasculature contributes to immune evasion mechanisms. Heterogeneous tumor vessels promote immune evasion by upregulating PD-L1 on both tumor and myeloid cells [12,31], that help in the recruitment of TAMs, MDSCs, and bone-marrow-derived cells, which in turn secrete pro-angiogenic cytokines including VEGF and CXCL12 [12,31]. Immunosuppressive M2-like TAMs, polarized by cytokine cues including IL-4 and IL-13 [122], are potent producers of VEGF, TGF-ꞵ, and MMP, all of which accelerate vessel formation and remodel the tissue to favor tumor progression [123]. In contrast, inflammatory M1-polarized macrophages, driven by stimuli including LPS and IFN-γ [124], tend to support vessel normalization and anti-tumor immunity [125], illuminating the dual and dynamic role of macrophage polarization states in shaping tumor angiogenesis and immunity.

In parallel, metabolic reprogramming is tightly coupled to angiogenesis in tumors. Hypoxia induces a metabolic shift toward aerobic glycolysis in both cancer cells and tumor endothelial cells, mediated by HIF-1 α-driven upregulation of glycolytic regulators such as PFKFB3 and GLUT1 [126,127]. Beyond supplying energy, glycolytic intermediates act as cofactors for chromatin-modifying enzymes, thereby linking cellular metabolism to the epigenetic activation of pro-angiogenic programs [128]. A similar mechanism operates in hyperglycolytic macrophages and microglia, which generate excess acetyl-coenzyme A that fuels histone acetylation and induces expression of pathological retinal angiogenesis–associated glycolytic macrophage/microglia (PRAGM) genes. This reprogramming enforces an angiogenic phenotype in immune cells, reinforcing vascular growth [129]. Furthermore, in a breast cancer model, although initial tumor regression was induced by anti-angiogenic multi-kinase inhibitors, tumors subsequently recurred despite suppressed angiogenesis. Gene expression profiling revealed a metabolic shift toward aerobic glycolysis and metabolic symbiosis through lactate transporters MCT1 and MCT4, demonstrating an adaptive mechanism that facilitates resistance to angiogenic blockade [120]. Together, such metabolic-epigenetic coupling drives tumor progression and metabolic reprogramming/symbiosis contributes to resistance against anti-angiogenic therapies.

**Figure 2:**
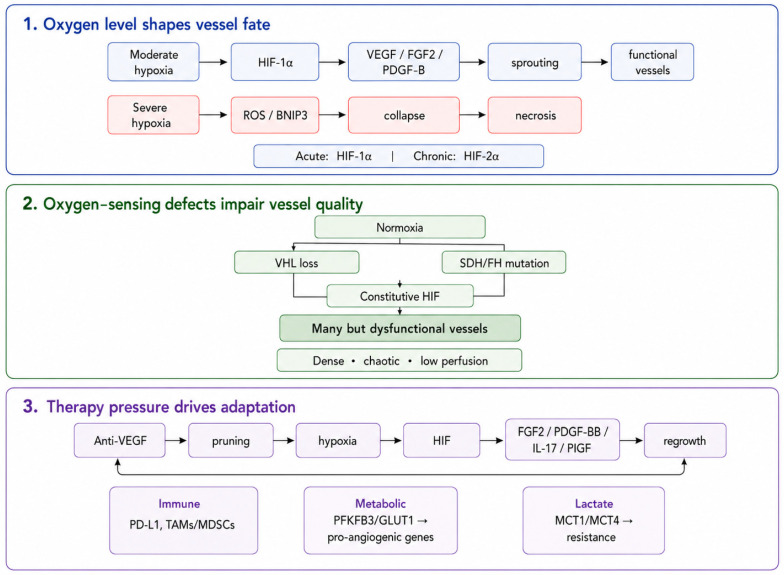
**Tumor vascular dynamics under hypoxic stress and therapeutic pressure.** Moderate hypoxia activates HIF-1α and pro-angiogenic signaling, promoting vessel sprouting and functional angiogenesis, whereas severe hypoxia activates ROS- and BNIP3-mediated stress pathways, leading to vascular collapse and necrosis. HIF-1α predominates during acute hypoxia, while HIF-2α supports longer-term vascular adaptation. Oxygen-sensing defects caused by VHL loss or SDH/FH mutations produce constitutive HIF activation and dense but dysfunctional, poorly perfused vessels. Anti-VEGF therapy can intensify hypoxia and HIF signaling, inducing compensatory pro-angiogenic factors and vascular regrowth; immune suppression, glycolytic reprogramming, and lactate shuttling further reinforce resistance to angiogenic blockade. Abbreviations: BNIP3, BCL2-interacting protein 3; FGF2, fibroblast growth factor 2; GLUT1, glucose transporter 1; IL-17, interleukin-17; MCT1/4, monocarboxylate transporters 1 and 4; MDSC, myeloid-derived suppressor cell; PDGF-B/BB, platelet-derived growth factor B/BB; PD-L1, programmed death-ligand 1; PFKFB3, 6-phosphofructo-2-kinase/fructose-2,6-bisphosphatase 3; PlGF, placental growth factor; ROS, reactive oxygen species; VEGF, vascular endothelial growth factor. Created in BioRender. https://BioRender.com/wvpa5is.

Tumor vascular behavior under hypoxic stress and therapeutic pressure is summarized in Figure 2.

## 5. Therapeutic Challenges in Targeting Angiogenesis

Clinical experience with anti-angiogenic agents indicates that VEGF-directed therapies typically induce only transient tumor responses and rarely achieve cures [18,130]. Although early preclinical studies predicted dramatic tumor regressions, clinical trials have shown that, in most cancers, adding VEGF inhibitors (like bevacizumab or VEGFR tyrosine-kinase inhibitors) extends median survival only modestly [130]. For example, in metastatic colorectal cancer the landmark Hurwitz trial found that adding bevacizumab to chemotherapy increased median overall survival to 20.3 months versus 15.6 months with chemo alone (a 4.7-month improvement) [131]. In glioblastoma and metastatic renal cell carcinoma (RCC), VEGF blockade prolongs progression-free survival (PFS) but has little effect on long-term overall survival [132,133]. Ultimately, most patients relapse due to the intrinsic or rapidly acquired resistance mechanisms that quickly re-establish tumor blood supply. This near-universal resistance demonstrates that while angiogenesis can be suppressed temporarily, tumors invariably adapt through multiple redundant and compensatory pathways.

### 5.1. Resistance to Anti-Angiogenic Therapies

Resistance originates via multiple, non-exclusive mechanisms. Tumors upregulate the compensatory pro-angiogenic factors such as FGF, PDGF, and ANGPTs, thereby restoring endothelial signaling despite VEGF blockade [134]. These signals can primarily originate from tumor cells themselves or from stromal elements like fibroblasts and infiltrating myeloid populations [135,136,137]. In glioblastoma, increased PDGF-C expression is associated with resistance to bevacizumab therapy, potentially contributing to alternative vascularization and tumor progression, but direct evidence for PDGF-C upregulation specifically following bevacizumab treatment in human tumors is limited [138]. Building on the alternative vascularization modes introduced in Section 2, two mechanisms, vascular co-option and vascular mimicry are particularly relevant to anti-angiogenic resistance. Vascular co-option allows tumor cells to hijack pre-existing vessels in highly perfused tissues like the brain and liver and bypasses the need for new vessel formation altogether [139]. This mechanism has been well characterized in brain metastases, advanced-stage NSCLCs and hepatocellular carcinoma, where tumors grow along native vasculature without inducing classical angiogenesis [139].

Vasculogenic mimicry, whereby tumor cells form perfusable, endothelial-like channels, further undermines anti-angiogenic efficacy [39]. These pseudo-vessels, seen in melanoma and glioblastoma, support perfusion independently of endothelial cells and correlate with poor prognosis [140,141]. VEGF-targeted therapy also induces hypoxia, stabilizing HIF-1α and activating downstream genes such as c-MET and β1 integrin to promote invasion, migration, and therapeutic resistance [142,143,144]. HIF-driven expression of VEGF and PD-L1 creates a feedback loop that reinforces angiogenesis and immunosuppression [145]. These processes are further supported by increased secretion of CXCL12 and TGF-β under hypoxia, which enhances tumor survival and stromal remodeling [146]. Thus, anti-angiogenic pressure remodels the tumor microenvironment to favor progression rather than regression.

### 5.2. Limitations of Current Strategies

Currently, anti-angiogenic therapies face critical limitations beyond biologic resistance. Clinical benefits are modest and often transient, with high rates of primary non-response across various tumor types [147]. For example, in metastatic colorectal cancer the landmark Hurwitz trial found that adding bevacizumab to chemotherapy increased median overall survival to 20.3 months versus 15.6 months with chemo alone (a 4.7-month improvement), but the majority of patients eventually relapse [131]. Similar patterns are observed in non-small cell lung cancer and glioblastoma, where initial radiographic responses do not consistently translate into prolonged survival [148,149]. A major barrier is the lack of robust biomarkers to guide therapy. Neither circulating VEGF levels nor tumor VEGF expression reliably predict response [150]. Imaging modalities such as DCE-MRI and PET tracers targeting hypoxia (e.g., 18F-FMISO) have been explored but remain investigational [151,152,153,154]. Without predictive markers, patients receive therapy empirically, leading to unnecessary exposure at a high cost.

Tumor heterogeneity also further undermines uniform targeting. Coexisting vascular phenotypes within a single lesion, ranging from classical angiogenesis to co-option and mimicry, suggest that VEGF-targeted agents only partially suppress tumor blood supply [155]. This spatial variability is evident in single-cell RNA sequencing datasets reflecting distinct endothelial subtypes with divergent responses to anti-angiogenic stress [33]. In preclinical models, high-dose anti-VEGF therapy reduced perfusion and hindered CD8+ T-cell infiltration, limiting synergy with immunotherapy [156,157]. Of note, this reduced perfusion can be therapeutically advantageous if medication is administered intratumorally, as observed with TKIs targeting VEGFR, where vascular regression leads to increased intratumoral drug concentrations [157]. These challenges underscore the need for refined approaches that link both vascular normalization with metabolic and immune modulation, guided by dynamic biomarkers and tailored to the heterogeneity of angiogenic dependencies.

## 6. Therapeutic Strategies to Modulate Hypoxic Pathways

Building upon these limitations, recent strategies have shifted toward targeting the hypoxic and pseudohypoxic circuitry that underlies angiogenic resilience. Emerging therapies are currently targeting the hypoxic and pseudohypoxic lifelines of tumors to overcome angiogenesis-driven progression and treatment resistance. These include direct inhibition of HIFs, hypoxia-activated prodrugs (HAPs), and vascular remodeling strategies that restore perfusion or modulate chemotactic gradients to improve drug delivery and immune access [158]. Hypoxic signaling is an effective axis for interaction as it not only drives vessel formation but also immune evasion, metabolic rewiring, and therapy resistance [159]. As further research is conducted about the tumor microenvironment, these strategies have shifted from broad anti-angiogenic paradigms to more nuanced approaches tailored to oxygen availability and immune infiltration status. The clinical success of such interventions will rely on precise biomarker integration, combination regimens, and optimized delivery.

### 6.1. HIF Inhibitors and Hypoxia-Activated Prodrugs

Belzutifan, a selective HIF-2α inhibitor, disrupts HIF-2α/ARNT dimerization and blocks transcription of *VEGF* and *EPO* [160]. In VHL-associated renal cell carcinoma, it demonstrated a 49% objective response rate (ORR), with responses also observed in pancreatic neuroendocrine tumors and hemangioblastomas at rates of 83% and 63% respectively [161]. Hypoxia and anemia, on-target effects due to EPO suppression, were common but manageable [162,163]. Other HIF-2α antagonists are in development, while HIF-1α inhibitors like PX-478 and IDF-11774 remain in preclinical or early-phase testing with limited translation so far [164,165,166].

HAPs such as evofosfamide (TH-302) release alkylating agents under hypoxia, showing efficacy in preclinical models [167]. However, in the MAESTRO phase III trial (NCT01746979) for pancreatic cancer, evofosfamide combined with gemcitabine did not significantly improve overall survival [168]. Tarloxotinib, a pan-HER HAP, also failed to show clinical benefit in head and neck squamous carcinoma [169]. Challenges with these agents include heterogeneous tumor oxygenation, poor penetration into hypoxic zones, and systemic toxicity. Newer agents such as CP-506 are in early-phase trials with improved activation thresholds (<1% O_2_) and are guided by hypoxia imaging and gene signatures for patient selection (NCT04954599) [170].

### 6.2. Vessel-Normalization vs. Decoy-Gradient Strategies

Rather than pruning tumor vessels, anti-angiogenic strategies can normalize vasculature to enhance oxygenation and drug delivery. This normalization window reduces interstitial pressure, facilitates immune cell infiltration, and suppresses HIF signaling. At appropriate doses, VEGF/VEGFR2 inhibition increases pericyte coverage and supports immune infiltration [171]. Clinically, combining anti-VEGF agents with checkpoint inhibitors has improved outcomes in hepatocellular and renal cell carcinomas [172]. However, overtreatment may lead to vascular collapse and hypoxia resurgence [173].

Alternatively, manipulating chemotactic gradients offers another route. SEMA3A can compete with VEGF at neuropilin-1 to repel aberrant vessels and reduce metastasis, despite excessive pruning leading to increased immunosuppression without immunotherapy [174]. CXCL12/CXCR4 signaling traps T cells at the tumor periphery and recruits suppressive myeloid cells. CXCR4 antagonists like balixafortide disrupt the trap and promote CD8+ T cell infiltration synergy with checkpoint blockade [175,176]. Trials combining motixafortide with pembrolizumab have shown early promise in resistant gastrointestinal tumors (NCT02826486). Together, vascular normalization and gradient-modulation are complementary approaches that remodel the hypoxic tumor microenvironment. Precision scheduling and biomarker guidance will be essential for integrating these therapies into combinatorial regimens.

### 6.3. Combination Therapies

Therapeutic strategies targeting hypoxia-driven angiogenesis are increasingly effective when deployed in combination with other modalities. Tumor hypoxia and pseudohypoxia activate parallel survival pathways and prompt an immunosuppressive microenvironment, meaning single-agent anti-angiogenic treatments often yield only transient benefits. Combining angiogenesis inhibitors with immunotherapy has yielded breakthrough results in multiple cancers. For example, adding the anti-VEGF antibody bevacizumab to PD-L1 blockade (e.g., atezolizumab) in unresectable hepatocellular carcinoma (HCC) improved median overall survival from 13.4 to 19.2 months compared to standard therapy (hazard ratio ~0.66, *p* < 0.001) [177]. Similarly in renal cell carcinoma (RCC), pairing a VEGF-targeted tyrosine kinase inhibitor with PD-1 checkpoint inhibition significantly increased objective response rates (60.6% vs. 39.6% with VEGF inhibition alone) [178]. These combinations exploit vessel normalization and immune reactivation: the anti-angiogenic component prunes aberrant vasculature and relieves tumor hypoxia, creating a window for immune cells or cytotoxic drugs to penetrate. Notably, preclinical studies confirm that radiotherapy administered during this “normalization window” achieves superior tumor control than outside it, explaining the rationale for combining angiogenesis blockade with radiation or chemotherapy to overcome hypoxia-induced resistance [179].

### 6.4. Biomarker-Guided Approaches and Patient Stratification

As multi-modal therapies expand, a critical challenge is identifying which patients will derive meaningful benefit from specific hypoxia-targeted strategies. Biomarker-guided stratification is emerging as the linchpin for personalizing angiogenesis-focused treatments. Historically, the absence of patient selection by tumor hypoxia status has been blamed for high-profile trial failures. For instance, hypoxia-activated prodrugs showed promise in early studies but faltered in phase III trials when given indiscriminately. Researchers argue that lack of stratification by tumor oxygenation was “sufficient to account for the failure” of these trials [180]. This lesson has spurred efforts to define robust hypoxia and pseudohypoxia biomarkers that can partition patients into responders and non-responders. One approach has been gene expression profiling to capture a tumor’s angiogenic and hypoxic signature. In lung cancer, a 20-gene expression signature reflecting high angiogenesis activity and low hypoxia identified patients who enjoyed markedly longer progression times under bevacizumab-based therapy (7.1 months vs. 2.1 months for low vs. high-risk patients respectively, *p* = 0.005). Those classified as “low hypoxia signature” also had a near doubling of overall survival (17.8 vs. 9.9 months) when treated with bevacizumab/erlotinib [181]. The outcomes of these signatures illustrate how biomarkers can predict therapeutic benefit and guide drug selection, sparing patients unlikely to respond from unnecessary prescription drugs.

In parallel, researchers are validating circulating and tissue markers tied to the hypoxic tumor microenvironment. Levels of HIFs and their downstream genes (e.g., *CA9*, *VEGF*, *ANGPT2*) in tumors or blood are being explored as predictive indicators. For example, in HCC patients treated with anti-angiogenic immunotherapy, a serum insulin-like growth factor (IGF-1) score was shown to stratify prognoses: patients with a favorable IGF-1 profile at baseline had approximately a 67% lower hazard of death than those with poor scores, independent of treatment (HR: 0.33) [182]. While prognostic in this case, such biomarker stratification could inform trial design and combination choices going forward. On the genomic front, recurrent *VHL* mutations in ccRCC exemplify a pseudohypoxic driver present in virtually all cases. This uniform biology underpins the exquisite sensitivity of these tumors to HIF-2α inhibitors [93]. In rarer settings, stratification may involve identifying metabolic hallmarks of pseudohypoxia, for instance succinate accumulation in *SDH*-mutant tumors or the oncometabolite 2-hydroxyglutarate (2-HG) in *IDH*-mutant cancers, to match patients with metabolism-targeted adjuncts. The overarching goal is to evolve beyond one-size-fits-all therapy and ground treatment decisions in the unique hypoxia/angiogenesis landscape of each tumor. As trials increasingly integrate biomarker analysis, the field is moving toward enriched designs where only “biomarker-positive” patients receive a hypoxia-targeted add-on [180]. This precision approach not only amplifies the observed benefit by treating those most likely to respond but also accelerates new approvals by showing clearer efficacy signals in stratified populations.

**Figure 3:**
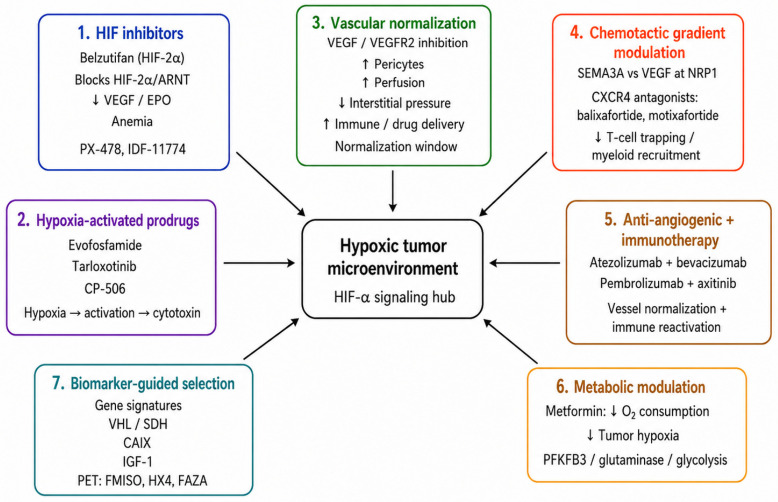
**Therapeutic strategies targeting the hypoxic tumor microenvironment.** Schematic overview of major therapeutic approaches directed at the hypoxic tumor microenvironment, organized around the central HIF-α signaling hub: (1) HIF inhibitors, including belzutifan (HIF-2α) and the investigational HIF-1α inhibitors PX-478 and IDF-11774; (2) hypoxia-activated prodrugs, including evofosfamide, tarloxotinib, and CP-506; (3) vascular normalization through VEGF/VEGFR2 inhibition to improve perfusion and immune/drug delivery; (4) chemotactic gradient modulation, including SEMA3A and CXCR4 antagonists (balixafortide and motixafortide); (5) combination anti-angiogenic therapy plus immunotherapy, including atezolizumab plus bevacizumab and pembrolizumab plus axitinib; (6) metabolic modulation, including metformin and inhibitors of PFKFB3, glutaminase, and glycolysis; and (7) biomarker-guided selection using hypoxia gene signatures, VHL/SDH mutational status, CAIX expression, serum IGF-1, and hypoxia PET imaging. Created in BioRender. https://BioRender.com/1rvhum1.

A remaining gap across these strategies is the scarcity of therapies that directly target vessel co-option, a major route of intrinsic and acquired resistance to anti-angiogenic agents [183,184]. Because co-opting tumor cells migrate along and hijack existing vessels rather than inducing new ones, they are largely insensitive to VEGF-pathway blockade. Emerging, mostly preclinical strategies therefore aim to disrupt the co-option machinery itself, including inhibition of tumor-cell motility (for example, actin-regulatory and Arp2/3-dependent pathways) [183], blockade of adhesion molecules such as L1CAM that mediate perivascular spreading [185], and modulation of the Ang2/Tie2 axis; combining such agents with anti-angiogenic therapy is being explored to prevent the co-option-driven escape that can follow vascular pruning [183,184]. To date, however, no anti-co-option agent has entered routine clinical use. These strategies are summarized in Figure 3.

## 7. Clinical Advances and Imaging Innovations

Recent therapeutic breakthroughs have begun to re-wire tumor lifelines by exploiting the hypoxic and pseudohypoxic vulnerabilities of cancers. A landmark example is the HIF-2α inhibitor belzutifan, which was FDA-approved in 2021 after demonstrating a 49% objective response rate in VHL syndrome-associated ccRCC [93]. In that pivotal trial, belzutifan also induced regression of highly vascular VHL-driven neoplasms, including pancreatic neuroendocrine tumors (77% response) and CNS hemangioblastomas (30% response) [93]. These responses, achieved with mostly low-grade toxicities (e.g., anemia in 90% of patients) [93], validate HIF targeting as a viable clinical strategy. Combinatorial regimens are further boosting efficacy: in a 2024 study, first-line belzutifan plus the VEGFR tyrosine kinase inhibitor cabozantinib yielded a 70% response rate (including 8% complete response) in metastatic ccRCC [107]. These results call attention to the translational payoff of neutralizing tumor “hypoxic drive,” especially in pseudohypoxic contexts like VHL disease or *SDH*-mutant tumors where HIFs are constitutively active. Belzutifan’s success in VHL-associated ccRCC has opened exploration of HIF-2α blockade in other pseudohypoxic neoplasms (e.g., hereditary pheochromocytomas), highlighting a new therapeutic paradigm [107].

Clinicians are now leveraging advanced imaging to guide these hypoxia-targeted therapies. Functional hypoxia imaging has matured into a clinical decision tool, with radiotracers like 18F-fluoromisonidazole (18F-FMISO) and newer analogs mapping intratumoral oxygen deficits in vivo. For instance, next-generation PET tracers such as 18F-flortanidazole (HX4) clear faster than FMISO, enabling sharper tumor-to-background contrast within 2.5 h of injection [186]. In a Phase I trial, 18F-HX4 exhibited no toxicities and produced hypoxia images with a median tumor-to-muscle ratio of 1.40 after 2 h [187]. These imaging innovations allow clinicians to stratify patients for hypoxia-specific treatments, a principle exemplified by trials combining hypoxia-activated prodrugs with PET guidance. In one study, the bioreductive drug evofosfamide (TH-302) added to radiotherapy significantly slowed tumor growth in xenografts; notably, only tumors with high pre-treatment HX4-PET uptake derived maximal benefit, suggesting hypoxia imaging can identify the patients most likely to respond [188]. Together, these clinical advances demonstrate a nascent but tangible transformation of hypoxia research into practice, where drugs and diagnostics co-evolve to erode the survival advantage conferred by tumor hypoxia.

### 7.1. Preclinical Studies

Multiple strategies derived through preclinical research converge on the HIF pathway, long deemed the master switch of hypoxic adaptation. For example, acriflavine, a drug identified to bind HIF-1α’s PAS-B domain, completely suppresses angiogenesis and induces near 100% long-term survival in orthotopic glioblastoma models when delivered via intracranial polymer wafers [189]. Acriflavine-treated tumors showed steep drops in VEGF, glycolytic enzymes, and perfusion-normalized growth kinetics, reflecting HIF-1’s role in its efficacy [189]. In parallel, silencing HIF expression has yielded synergistic benefits when combined with immunotherapy [190]. These findings align with emerging evidence that hypoxia fosters immune escape; thus, “normalizing” tumor oxygen or HIF activity can revitalize anti-tumor immunity. Consistent with this concept, strategies to normalize aberrant tumor vasculature are gaining traction. A recent study identified LRG1 (leucine-rich a-2-glycoprotein 1) as a novel vessel destabilizer upregulated in solid tumors; genetic or antibody-mediated LRG1 blockade yielded more mature, pericyte-covered vessels, improving perfusion and cutting hypoxic fractions in mice [191]. These preclinical insights illustrate how reconditioning the tumor microvasculature can convert a vicious cycle of hypoxia-driven resistance into a cycle of improved drug delivery.

Innovative hypoxia-activated prodrugs are similarly showing promise in preclinical trials. CP-506 is a next-generation nitroaromatic prodrug designed to release a DNA-crosslinking toxin specifically under hypoxia. In murine models of head and neck cancer, CP-506 by itself induced substantial DNA damage and tumor control in hypoxic tumors, and when combined with a single dose of radiation, the local cure rate more than doubled (62% vs. 27% with radiation alone, *p* = 0.024) [170]. This debulking of hypoxic zones can have a two-fold benefit: directly killing therapy-resistant cells and secondarily reoxygenating the tumor to improve subsequent therapies (as reflected by CP-506’s greater impact under hypofractionated radiation, which exploits residual hypoxia, vs. conventional fractionation) [170]. The strong preclinical efficacy of CP-506 has fast-tracked it into a first-in-human Phase 1/2 trial (NCT04954599). On another front, researchers are developing next-generation imaging probes to visualize and quantify tumor hypoxia with unprecedented precision. One breakthrough is the development of a PET tracer that directly targets HIF-2α, enabling noninvasive imaging of HIF activity rather than oxygen proxies. A prototype agent, [^18^F]-TC-S 7009, was recently synthesized by radiolabeling a selective HIF-2α antagonist (K_d_ = 81 nM) [192]. In glioblastoma-bearing mice, [^18^F]-TC-S 7009 penetrated the blood–brain barrier and accumulated in tumors with high HIF-2 levels, achieving clear tumor-to-background contrast within 60 min [192]. Beyond PET, multimodal imaging techniques are being explored to capture the dynamic landscape of tumor oxygenation. Photoacoustic imaging, for example, can real-time visualize oxygen saturation at high spatial resolution, either label-free (via endogenous hemoglobin spectra) or with exogenous contrast agents that “turn on” in hypoxic conditions [193]. Likewise, fluorescent theranostic probes are in development (e.g., NIR-emitting nanoparticles that become phosphorescent in low pO_2_, which can simultaneously image hypoxia and deliver cytotoxic payloads once activated [194].

### 7.2. Clinical Trials

Translating the hypoxia and pseudohypoxia paradigm into successful therapies has proved challenging, but recent trials are probing multiple strategies, as outlined in Table 2. HAPs remain an enticing but challenging approach. The lead HAP evofosfamide (TH-302) releases a DNA alkylator in oxygen-starved zones. While it improved progression-free survival in a Phase II pancreatic cancer study (when added to gemcitabine) (NCT01144455) [168], the pivotal MAESTRO Phase III trial in advanced pancreas cancer failed to improve overall survival with evofosfamide plus gemcitabine versus chemotherapy alone [195]. Similarly, tarloxotinib, a prodrug that releases a pan-ErbB tyrosine kinase inhibitor under hypoxia, showed minimal benefit in a Phase II study for refractory or metastatic head and neck cancer (ORR 3% and no PFS improvement) (NCT02449681) [169]. Nonetheless, next-generation HAPs are under evaluation. For example, CP-506 is a nitroimidazole prodrug optimized to trigger below 1% O_2_ and is being tested with patient selection based on hypoxia biomarkers (hypoxia gene-expression signatures and PET imaging) in an ongoing trial (NCT04954599).

Hypoxia-driven angiogenesis often leads to abnormal vasculature and an immunosuppressive microenvironment, so combining vascular normalization with immune activation is a logical strategy. Multiple trials have now validated this approach. In unresectable hepatocellular carcinoma, the VEGF-neutralizing antibody bevacizumab plus anti–PD-L1 immunotherapy (atezolizumab) achieved superior outcomes over standard therapy (sorafenib). The IMbrave150 trial reported an ORR of 27.3% vs. 11.9% and median PFS of 6.8 vs. 4.3 months, with a 42% reduction in risk of death (HR 0.58) for the combination therapy (NCT03434379) [196]. Notably, 5.5% of these patients had complete responses under the combination [196]. An updated analysis of IMbrave150 with 12 additional months of follow-up (median 15.6 months) confirmed and strengthened these findings: median overall survival was 19.2 vs. 13.4 months (HR 0.66; 95% CI 0.52–0.85; *p* < 0.001), median PFS was 6.9 vs. 4.3 months (HR 0.65; 95% CI 0.53–0.81; *p* < 0.001), and the ORR by RECIST 1.1 increased to 30% with an 8% complete response rate. The safety profile remained consistent with the primary analysis, with treatment-related grade 3/4 adverse events in 43% vs. 46% of patients [177], and the median time to deterioration of quality of life was markedly longer with the combination (11.2 vs. 3.6 months) [197]. These updated results cemented atezolizumab plus bevacizumab as the first-line standard of care for advanced hepatocellular carcinoma.

Beyond conventional anti-angiogenic and immunotherapy combinations, emerging efforts are exploring metabolic modulation of the hypoxic tumor microenvironment. A Phase II randomized trial (NCT04114136) is recruiting patients with advanced solid tumors to evaluate anti-PD-1 therapy (pembrolizumab or nivolumab) combined with metabolic modulators (metformin or rosiglitazone). The rationale is grounded in preclinical evidence that metformin can reduce tumor hypoxia by decreasing mitochondrial oxygen consumption, thereby increasing intratumoral oxygen availability and potentially enhancing immune checkpoint blockade efficacy.

In parallel, several ongoing trials are advancing hypoxia-targeted imaging to guide patient selection for hypoxia-directed therapies. These include evaluation of 18F-EF5 PET/CT for noninvasive quantitative mapping of intratumoral hypoxia (NCT04001023), integrated PET-MRI to combine metabolic and functional imaging for comprehensive characterization of the hypoxic microenvironment (NCT05246475), and 18F-FAZA PET for hypoxia imaging in lung cancer, leveraging this second-generation tracer’s improved pharmacokinetics and higher tumor-to-background contrast relative to 18F-FMISO (NCT02701699). Additionally, a trial (NCT06455761) is investigating the diagnostic performance of 68Ga-DOTA-NI-FAPI04 PET/CT across multiple cancer types, comparing results with 68Ga-FAPI and 18F-FDG PET/CT; this dual-target approach combines fibroblast activation protein inhibitor targeting with nitroimidazole-based hypoxia sensing, enabling simultaneous assessment of stromal activation and tumor hypoxia in a single session. Separately, a trial (NCT06796764) is evaluating the COMBO Endoscopy Oropharyngeal Airway device versus standard nasal cannula for optimizing oxygenation monitoring during endoscopic procedures in cancer patients.

**Table 2:** Clinical trials targeting hypoxia and angiogenesis-driven pathways in cancer. Trials are organized by therapeutic category: hypoxia-activated prodrugs (HAPs), HIF inhibitors, anti-angiogenic/immunotherapy combinations, metabolic modulators, and hypoxia-targeted imaging strategies. Outcome status is denoted as positive (improved endpoint met), negative (primary endpoint not met), or ongoing. Abbreviations: ccRCC, clear cell renal cell carcinoma; CR, complete response; GI, gastrointestinal; H&N, head and neck; HAP, hypoxia-activated prodrug; HCC, hepatocellular carcinoma; ICB, immune checkpoint blockade; mOS, median overall survival; ORR, objective response rate; PFS, progression-free survival; pNET, pancreatic neuroendocrine tumor; QoL, quality of life; RCC, renal cell carcinoma; SoC, standard of care; TKI, tyrosine kinase inhibitor; TME, tumor microenvironment. Trials in the MANGO trial and Phase I HX4 rows are additional trials identified during manuscript preparation (not cited in Section 7.2 main text).

Trial/Identifier	Tumor Type	Intervention	Phase	Key Outcome	Status/Notes
Hypoxia-Activated Prodrugs (HAPS)					
NCT01144455	Advanced pancreatic cancer	Evofosfamide (TH-302) + gemcitabine	II	Improved PFS vs gemcitabine alone	Preceded Phase III MAESTRO
MAESTRO (NCT01746979)	Advanced pancreatic cancer	Evofosfamide (TH-302) + gemcitabine	III	No improvement in OS vs. chemo alone	Lack of hypoxia stratification cited as key limitation
NCT02449681	Recurrent/ metastatic head and neck squamous cell carcinoma	Tarloxotinib (pan-ErbB HAP)	II	ORR 3%; no PFS improvement	Minimal benefit; trial closed
NCT04954599	Solid tumors (including H&N)	CP-506 (next-gen nitroimidazole; <1% O_2_ threshold) ± radiation; guided by hypoxia PET and gene signatures	I/II	Ongoing; first-in-human	Biomarker- selected enriched design
HIF Inhibitors					
MK-6482-004 [93]	VHL-associated ccRCC, pNET, hemangioblastoma	Belzutifan (MK-6482; HIF-2α inhibitor; FDA approved 2021)	II	ORR 49% ccRCC; 83% pNET; 63% hemangioblastoma	Led to FDA approval; anemia in ~90%
LITESPARK-003 (NCT03634540)	Metastatic ccRCC (1st line)	Belzutifan + cabozantinib (VEGFR TKI)	II	ORR 70% (8% CR)	2024 data; combination benchmark
Combination: Anti-Angiogenic + Immunotherapy					
IMbrave150 (NCT03434379)	Unresectable hepatocellular carcinoma (HCC)	Atezolizumab (anti–PD-L1) + bevacizumab (anti-VEGF) vs. sorafenib	III	mOS 19.2 vs. 13.4 mo (HR 0.66); ORR 30%; 8% CR; now 1st-line SoC	Updated analysis confirmed benefit; QoL superior
KEYNOTE-426	Advanced RCC (1st line)	Pembrolizumab (anti–PD-1) + axitinib (VEGFR TKI) vs. sunitinib	III	ORR 59.3% vs. 35.7%; durable at 43-mo follow-up	Established combination paradigm in RCC
NCT02826486	Resistant GI tumors	Motixafortide (CXCR4 antagonist) + pembrolizumab	I/II	Early promise reported; ongoing	CXCR4 disrupts T-cell trapping at tumor periphery
Metabolic Modulation of Hypoxic Time					
NCT04114136	Advanced solid tumors	Anti–PD-1 (pembrolizumab or nivolumab) + metformin or rosiglitazone	II	Recruiting; primary endpoints pending	Metformin reduces mitochondrial O_2_ consumption → may enhance ICB efficacy
Hypoxia-Targeted Imaging and Patient Stratification					
NCT04001023	Solid Tumors	^18^F-EF5 PET/CT for noninvasive quantitative hypoxia mapping	—	Ongoing	Quantitative intratumoral hypoxia characterization
NCT05246475	Solid Tumors	Integrated PET-MRI (metabolic + functional hypoxia imaging)	—	Ongoing	Comprehensive TME characterization
NCT02701699	Lung Cancer	^18^F-FAZA PET (2nd-gen tracer; improved pharmacokinetics vs. ^18^F-FMISO)	—	Ongoing	Higher tumor-to-background contrast than FMISO
NCT06455761	Multiple tumor types	^68^Ga-DOTA-NI-FAPI04 PET/CT vs. ^68^Ga-FAPI + ^18^F-FDG PET/CT (dual-target: stromal + hypoxia)	—	Ongoing	Single-session stromal activation + hypoxia assessment
NCT06796764	Cancer patients (endoscopy)	COMBO Endoscopy Oropharyngeal Airway device vs. standard nasal cannula for oxygenation monitoring	—	Ongoing	Procedural oxygenation optimization; not hypoxia-directed therapy per se
MANGO trial [198]	Glioblastoma	OE-MRI + BOLD MRI vs. ^18^F-FMISO PET for radiotherapy planning	—	Ongoing	MRI biomarker validation vs. gold-standard PET hypoxia imaging
Phase I HX4 [187]	Solid tumors	^18^F-HX4 (flortanidazole) PET; faster clearance, higher contrast than FMISO	—	No toxicities; tumor-to-muscle ratio 1.40 at 2 h; good test–retest reproducibility	Used in evofosfamide combination studies

### 7.3. Advanced Imaging

Advances in imaging have empowered clinicians to noninvasively map tumor hypoxia and angiogenic status with unprecedented detail to guide therapy [199]. Several hypoxia-specific PET tracers have been developed to visualize oxygen deprivation in vivo [200]. The prototypical agent [^18^F]-fluoromisonidazole ([^18^F]-FMISO), selectively accumulates in cells with <10 mmHg oxygen and has been used in many clinical studies to delineate hypoxic tumor subregions [201]. FMISO-PET is highly specific for hypoxia, but its slow clearance from normoxic tissue can yield modest contrast images [202]. To further expand on this, “second-generation” tracers like [^18^F]-FAZA and [^18^F]-HX4 were designed for greater hydrophilicity and faster washout [203]. However, some candidates like [^18^F]-FETNIM did not outperform FMISO in clinical comparison, hinting that tracer behavior can vary by tumor type [204]. Among the promising next-generation agents is [^18^F]-flortanidazole ([^18^F]-HX4) with a more favorable pharmacokinetic profile (clearance half-life ~1–2 h) and greater hydrophilicity than FMISO, resulting in higher-contrast images of hypoxic zones [186]. Early-phase trials have confirmed HX4’s safety and feasibility in patients and demonstrated a good test–retest reproducibility of hypoxia measurements [187]. In a representative analysis, pre-treatment FMISO PET tumor-to-muscle ratios and tumor-to-mediastinum ratios in non-small cell lung cancer and head-neck cancers significantly predicted poorer local control after radiotherapy. All patients with tumor-to-mediastinum ratios > 2.0 (NSCLC) or tumor-to-muscle ratios > 1.6 (head-neck cancer) experienced tumor recurrence within one year, whereas only 27% of patients below these thresholds had recurrence. Additionally, accumulation-type kinetic curves on dynamic FMISO imaging were highly predictive of local recurrence [205]. Such findings bolster the use of hypoxia PET for risk stratification and for adaptive therapy [206].

In addition to PET, innovative MRI techniques now allow visualization of tumor oxygenation and vascular function without exogenous tracers [207]. Blood-Oxygen-Level-Dependent (BOLD) MRI exploits the paramagnetism of deoxyhemoglobin as an intrinsic contrast [199]. In one pilot study, BOLD MRI signal intensity in a rodent tumor model showed a significant inverse correlation with FMISO PET uptake (*p* = 0.003), confirming that regions with low BOLD (high deoxy-Hb) coincided with high hypoxia tracer retention [208]. Clinically, oxygen-enhanced MRI (OE-MRI) measures T_1_ signal changes resulting from dissolved oxygen entering tissues and is often combined with BOLD MRI to improve estimation of tissue oxygenation [209]. For example, the ongoing MANGO trial is simultaneously assessing OE-MRI and BOLD MRI against FMISO PET in glioblastoma patients to validate MRI biomarkers of hypoxia for radiotherapy planning [198]. A powerful emerging concept is combining imaging of tumor vasculature and hypoxia to capture the full picture of angiogenic status. One approach is sequential multi-tracer PET. For example, a patient can undergo [^15^O]-water PET to measure perfusion, immediately followed by [^18^F]-FMISO PET to assess hypoxia, leveraging the short half-life of ^15^O [210]. Another strategy is dual-target tracers that probe two aspects of the tumor microenvironment in one molecule [211]. A recent example is a PET agent coupling a fibroblast activation protein inhibitor and an RGD-peptide (α_vβ_3_ integrin ligand) in a single radiotracer [212]. Such dual imaging can highlight tumors that are simultaneously metabolically active and highly vascular, versus those with a metabolic–vascular mismatch (e.g., high glycolysis but low blood flow, which often indicates diffusion-limited hypoxia). Going forward, accuracy metrics for these advanced techniques are being rigorously evaluated such as the test–retest variability of hypoxia PET (~15% for volume metrics) sets a baseline for meaningful change. Recent studies report that BOLD MRI has variable reproducibility for R2 measurements in tumors, with test–retest coefficients of variation ranging from <15% at 1.5 T to <35% at 3.0T in hepatocellular carcinoma [213], and a coefficient of repeatability of 11.2 ms for median T2 values in colorectal liver metastases [214]. Each technical advance thus directly feeds into better clinical decision-making for angiogenesis-driven cancers, ensuring that hypoxia no longer remains a “black box” obstacle but rather a measurable, targetable facet of the patient’s disease. Advanced imaging can also help flag non-angiogenic, vessel co-opting tumors: because these lesions grow along pre-existing vasculature and lack a florid neovascular signature, they may appear deceptively low on angiogenesis-focused perfusion imaging [92,185]. On perfusion MRI, for example, co-option–dominant lesions may show only modest elevation of relative cerebral blood volume, relatively low vascular permeability, and limited response to VEGF blockade [185]. Recognizing this vascular pattern is important for identifying patients unlikely to benefit from anti-angiogenic therapy.

## 8. Conclusions and Future Directions

Hypoxia and pseudohypoxia emerge from this analysis as pivotal yet paradoxical regulators of tumor vascularization and therapy responsiveness. Both true oxygen deprivation and oxygen-independent HIF stabilization (e.g., via VHL or SDH mutations) potently drive pro-angiogenic, metabolic, and immune-reprogramming programs that fuel malignancy progression and treatment resistance. Notably, hypoxic signaling is context-dependent. While moderate hypoxia induces VEGF-led neovascularization, in extreme cases oxygen deprivation can collapse vascular networks instead of sprouting new ones. Likewise, pseudohypoxia recapitulates a “HIF-ON” state even in normoxia, sustaining Warburg metabolism and neovascular signals akin to chronic hypoxia. In other words, hypoxia and pseudohypoxia “rewire” tumor lifelines through convergent HIF pathways, but the downstream consequences are complex, necessitating equally sophisticated therapeutic strategies. Recognition of angiogenic plasticity and hypoxic remodeling has spurred a shift from one-dimensional VEGF blockade toward multipronged interventions. Importantly, hypoxia-induced immunosuppression is now recognized as a major resistance node: VEGF and low oxygen tension promote accumulation of regulatory T cells and M2-like macrophages while excluding cytotoxic T lymphocytes. This provides a rationale for combining angiogenesis inhibitors with immunotherapies. Looking forward, tackling angiogenic plasticity will likely require such multi-modal regimens that concurrently target blood vessel growth, hypoxia signaling, and immune dysfunction, rather than any single pathway in isolation.

Several translational breakthroughs are poised to exploit the hypoxic Achilles’ heel of tumors. The first-in-class HIF-2α inhibitor belzutifan (MK-6482) exemplifies the therapeutic potential of directly targeting pseudohypoxic drives: in von Hippel–Lindau patients with renal cell carcinoma, belzutifan achieved durable tumor regression (objective response of 49%) by silencing the HIF-2 oncogenic program, leading to its recent FDA approval. Ongoing phase III trials will reveal whether such HIF-targeted strategies can broaden to sporadic cancers, marking a new paradigm of “starving” tumors of their hypoxic survival signals. Another approach rekindled by recent research is hypoxia-activated prodrugs (HAPs). Agents like evofosfamide (TH-302) showed selective toxicity in hypoxic tumor zones, though prior late-stage trials failed to improve outcomes, partly due to patient heterogeneity in tumor oxygenation. Moreover, tumor cells in hypoxic niches accumulate lactate and adenosine that paralyze effector T cells and NK cells while energizing immunosuppressive regulatory T cells and macrophages. To counter this, innovative therapies are aiming beyond the endothelial compartment. For example, inhibitors of carbonic anhydrase IX, lactate dehydrogenase, or adenosine A2A receptors are being tested to metabolically re-educate the tumor immune microenvironment toward anti-tumor activity [215,216,217]. This opens the door to trimodality regimens, comprising anti-VEGF, checkpoint inhibitor, and metabolic modulator therapies, reflecting a holistic attack on the hypoxic tumor niche on multiple fronts.

In sum, the future of targeting hypoxia-driven angiogenesis lies in rational combination and precision medicine. Therapeutic targeting of hypoxia pathways, angiogenic escape routes, and tumor immune metabolism should be orchestrated based on robust biomarkers that identify each tumor’s dependence on these processes. Advances in hypoxia imaging and “omics” profiling will allow clinicians to stratify patients and dynamically monitor treatment responses, ensuring that aggressive interventions (HIF inhibitors, HAPs, multi-target angiokinase inhibitors, etc.) are deployed only when a hypoxic signature is present. Meanwhile, continued basic research is imperative to resolve unanswered questions, for example, determining the role of lesser-studied hypoxia regulators (like HIF-3α or oxygen-sensing mitochondria) and the optimal timing to combine vascular normalization with immune therapy. By embracing the complexity of hypoxia and pseudohypoxia rather than oversimplifying it, we can convert these adaptive tumor lifelines into liabilities. The next generation of trials will test highly tailored interventions that simultaneously disable hypoxic signaling, thwart angiogenic redundancy, and recalibrate immune-metabolic circuits in tumors. Such an integrated strategy holds the promise of finally breaking through the longstanding therapeutic impasse.

## Data Availability

No new data were created or analyzed in this study. Data sharing is not applicable to this article.
